# Crystal structure of *N*-[(8*E*)-12-methyl-14-phenyl-10,13,14,16-tetra­aza­tetra­cyclo­[7.7.0.0^2,7^.0^11,15^]hexa­deca-1(16),2,4,6,9,11(15),12-heptaen-8-yl­idene]hydroxyl­amine 1,4-dioxane hemisolvate

**DOI:** 10.1107/S2056989014027285

**Published:** 2015-01-03

**Authors:** Shaaban K. Mohamed, Joel T. Mague, Mehmet Akkurt, Talaat I. El-Emary, Mustafa R. Albayati

**Affiliations:** a Chemistry and Environmental Division, Manchester Metropolitan University, Manchester M1 5GD, England; b Chemistry Department, Faculty of Science, Minia University, 61519 El-Minia, Egypt; c Department of Chemistry, Tulane University, New Orleans, LA 70118, USA; d Department of Physics, Faculty of Sciences, Erciyes University, 38039 Kayseri, Turkey; e Department of Chemistry, Faculty of Science, Assiut University, 71515 Assiut, Egypt; f Kirkuk University, College of Science, Department of Chemistry, Kirkuk, Iraq

**Keywords:** crystal strcuture, pyrazino­pyrazoles, oximes, hydrogen bonding, π–π stacking

## Abstract

In the title solvate, C_19_H_13_N_5_O·0.5C_4_H_8_O_2_, the main mol­ecule is almost planar (r.m.s. deviation for the non-H atoms = 0.066 Å). The hydroxyl­amine group is disordered over two orientations in a 0.761 (4):0.239 (4) ratio. The complete dioxane solvent mol­ecule is generated by a crystallographic inversion centre. In the crystal, both disorder components of the hydroxyl­amine group form O—H⋯N hydrogen bonds to the same N-atom acceptor, thereby generating [010] chains. The chains encompass [010] channels occupied by the solvent mol­ecules. Aromatic π–π stacking is also observed [shortest centroid–centroid separation = 3.3394 (19) Å].

## Related literature   

For a related structure see: Mague *et al.* (2014 ▸). For background to the biological properties of pyrazino­pyroles or pyrazino­pyrazoles see: Nyeki *et al.* (2002 ▸); Askew *et al.* (1997 ▸); Wehner *et al.* (1998 ▸); Zimmerman (1995 ▸).
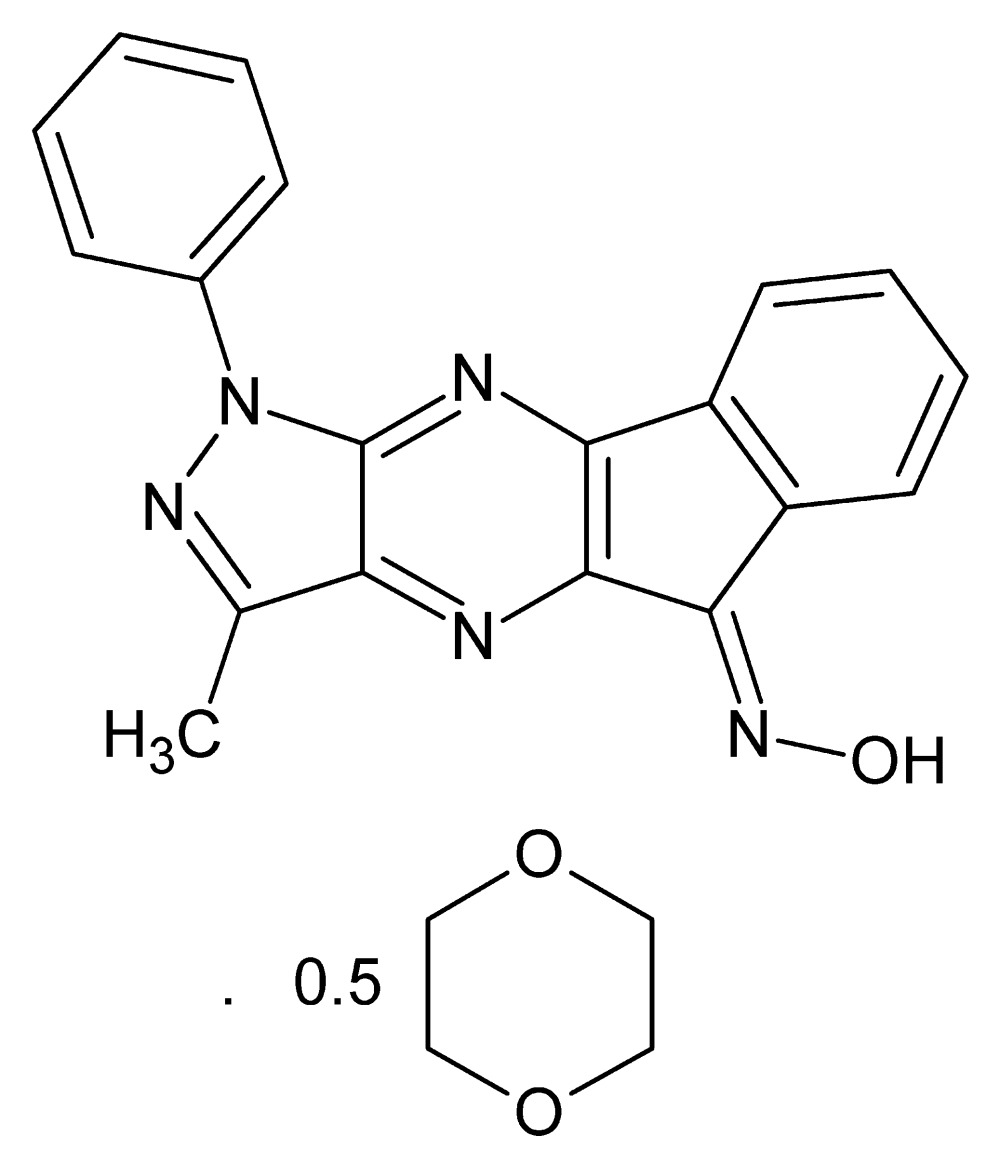


## Experimental   

### Crystal data  

C_19_H_13_N_5_O·0.5C_4_H_8_O_2_
*M* *_r_* = 371.40
Monoclinic, 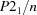

*a* = 15.8019 (4) Å
*b* = 5.5675 (1) Å
*c* = 20.4756 (5) Å
β = 102.093 (2)°
*V* = 1761.41 (7) Å^3^
*Z* = 4
Cu *K*α radiation
μ = 0.77 mm^−1^
*T* = 150 K
0.15 × 0.07 × 0.04 mm

### Data collection  

Bruker D8 VENTURE PHOTON 100 CMOS diffractometer
Absorption correction: multi-scan (*SADABS*; Bruker, 2014 ▸) *T*_min_ = 0.85, *T*_max_ = 0.97
12942 measured reflections
3122 independent reflections
1934 reflections with *I* > 2σ(*I*)
*R*_int_ = 0.066

### Refinement  

*R*[*F* ^2^ > 2σ(*F* ^2^)] = 0.060
*wR*(*F* ^2^) = 0.166
*S* = 1.05
3122 reflections
261 parameters
2 restraints
H-atom parameters constrained
Δρ_max_ = 0.49 e Å^−3^
Δρ_min_ = −0.21 e Å^−3^

### 

Data collection: *APEX2* (Bruker, 2014 ▸); cell refinement: *SAINT* (Bruker, 2014 ▸); data reduction: *SAINT*; program(s) used to solve structure: *SHELXT* (Sheldrick, 2008 ▸); program(s) used to refine structure: *SHELXL2014* (Sheldrick, 2008 ▸); molecular graphics: *DIAMOND* (Brandenburg & Putz, 2012 ▸); software used to prepare material for publication: *SHELXTL* (Sheldrick, 2008 ▸).

## Supplementary Material

Crystal structure: contains datablock(s) global, I. DOI: 10.1107/S2056989014027285/hb7340sup1.cif

Structure factors: contains datablock(s) I. DOI: 10.1107/S2056989014027285/hb7340Isup2.hkl

Supporting information file. DOI: 10.1107/S2056989014027285/hb7340Isup3.cml

**Fig. 1.**
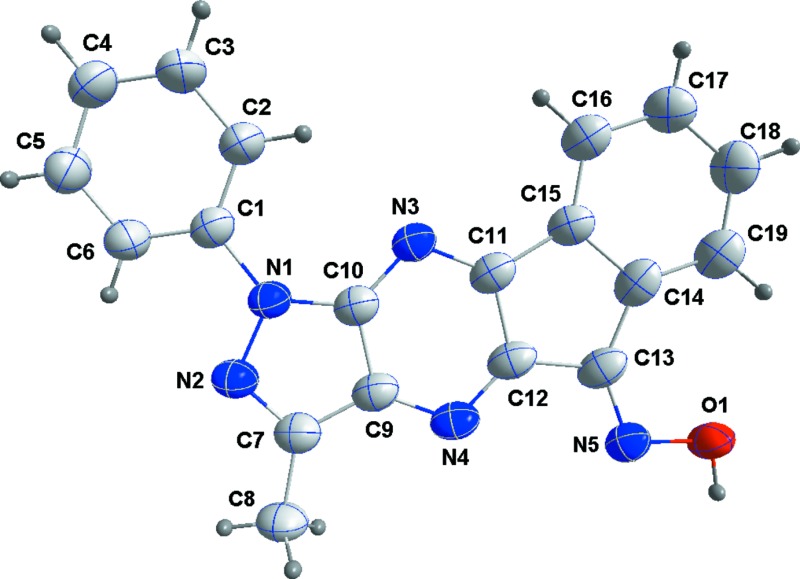
The title molecule showing 50% probability ellipsoids. Only the major componenet of the disordered hydroxylamine substituent is shown.

. DOI: 10.1107/S2056989014027285/hb7340fig1.tif
The title mol­ecule showing 50% probability ellipsoids. Only the major componenet of the disordered hydroxyl­amine substituent is shown.

**Fig. 2.**
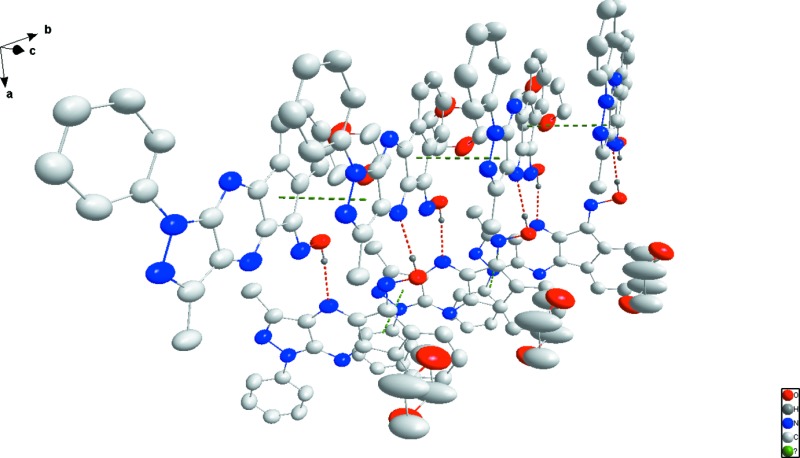
Packing showing the π-π interactions as green dotted lines.

**Fig. 3.**
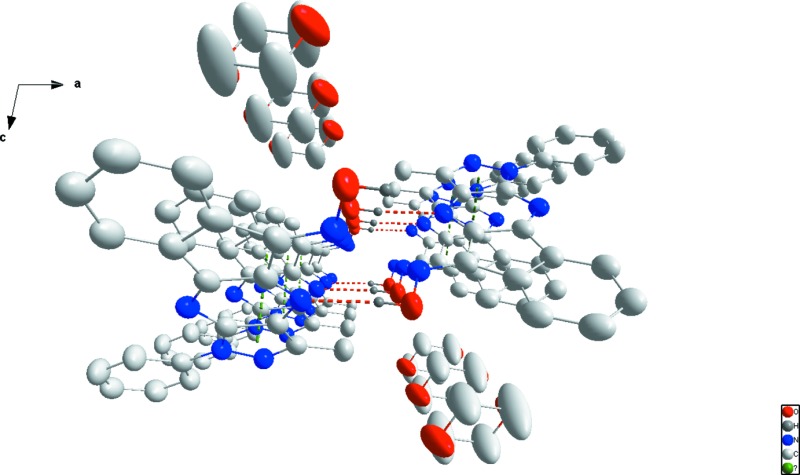
Packing viewed down the b axis showing the formation of one column via O—H···N hydrogen bonds (red dotted lines).

. DOI: 10.1107/S2056989014027285/hb7340fig2.tif
Packing showing the π-π inter­actions as green dotted lines.

b via . DOI: 10.1107/S2056989014027285/hb7340fig3.tif
Packing viewed down the *b* axis showing the formation of one column *via* O—H⋯N hydrogen bonds (red dotted lines).

CCDC reference: 1039120

Additional supporting information:  crystallographic information; 3D view; checkCIF report

## Figures and Tables

**Table 1:** Hydrogen-bond geometry (, )

*D*H*A*	*D*H	H*A*	*D* *A*	*D*H*A*
O1H1N4^i^	0.84	2.04	2.878(3)	172
O1aH1aN4^i^	0.84	1.94	2.749(5)	162

Symmetry code: (i) 
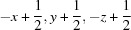
.

## supplementary crystallographic information

### S1. Comment

Heterocyclic compounds containing pyrolo- or pyrazino-pyrazole core structures represent a relatively little-explored group with interesting pharmaceutical properties. They have been described as vasodilators (Nyeki *et al.*, 2002), fibrinogen receptor antagonists with antiplatelet activity (Askew *et al*., 1997), vitronectin-receptor antagonists (Wehner *et al.*, 1998) and herbicidal agents (Zimmerman, 1995). In a continuation of our efforts towards the synthesis of bio-active pyrazinopyrazines, we report here the synthesis and crystal structure of the title compound.

The fused, four-ring core of the title molecule (Fig. 1) is nearly planar with only a 3.0 (2)° dihedral angle between the C14–C19 and C7/C9/C10/N1/N2 rings while the dihedral angle between the latter ring and the pendant phenyl ring is 5.2 (2)°. The values of the geometric parameters of the title molecule are normal and are comparable to those reported for a similar structure (Mague *et al.*, 2014).

The molecules form stacks *via*π-π interactions between the C7/C9/C10/N1/N2 ring in one molecule with the C11–C15 ring in the molecule at *x*, -1 + *y*, *z* (centroid–centroid distance = 3.34 Å, Fig. 2). Two screw-axis-related stacks are associated *via* O1—H1···N4 hydrogen bonds forming columns running parallel to the *b* axis (Table 1 and Fig. 3). In each column, the mean planes of the molecules in one stack are inclined to those of the second by 73.3°. The solvent dioxane molecules lie adjacent to the hydroxylamine groups and fill channels between the columns.

### S2. Experimental

A mixture of 2 mmol (624 mg) of 3-methyl-1-phenylindeno[2,1-*e*]pyrazolo[3,4-*b*]pyrazin-5(1*H*)-one and 2 mmol (139 mg) of hydroxylamine hydrochloride in dry pyridine (15 ml) was heated under reflux for 3 h. After cooling, the reaction mixture was poured into an ice-water mixture. The resulting solid product was then filtered off, washed with water, dried and crystallized from a mixture of dioxane/water (1:2 *v*/*v*) to afford light yellow crystals of the title compound. Mp 577 – 579 K.

### S3. Refinement

H-atoms attached to carbon atoms were placed in calculated positions (C—H = 0.95 - 0.98 Å) while that attached to the oxygen atom was placed in a location derived from a difference map and its parameters adjusted to give O—H = 0.84 Å. All were included as riding contributions with isotropic displacement parameters 1.2 - 1.5 times those of the attached atoms. The {N—OH} unit is disordered over two resolved sites in a 3:1 ratio and was refined subject to restraints that the geometries of the two components be comparable. The solvent molecule of dioxane located on a center of symmetry appeared to be slightly disordered on the basis of the size and shape of its displacement ellipsoids but attempts to refine it with a split atom model were unsuccessful.

### Crystal data

**Table:**

C_19_H_13_N_5_O·0.5C_4_H_8_O_2_	*F*(000) = 776
*M**_r_* = 371.40	*D*_x_ = 1.401 Mg m^−^^3^
Monoclinic, *P*2_1_/*n*	Cu *K*α radiation, λ = 1.54178 Å
*a* = 15.8019 (4) Å	Cell parameters from 5735 reflections
*b* = 5.5675 (1) Å	θ = 3.2–67.0°
*c* = 20.4756 (5) Å	µ = 0.77 mm^−^^1^
β = 102.093 (2)°	*T* = 150 K
*V* = 1761.41 (7) Å^3^	Column, light yellow
*Z* = 4	0.15 × 0.07 × 0.04 mm

### Data collection

**Table:**

Bruker D8 VENTURE PHOTON 100 CMOS diffractometer	3122 independent reflections
Radiation source: INCOATEC IµS micro-focus source	1934 reflections with *I* > 2σ(*I*)
Mirror monochromator	*R*_int_ = 0.066
Detector resolution: 10.4167 pixels mm^-1^	θ_max_ = 67.1°, θ_min_ = 3.2°
ω scans	*h* = −18→18
Absorption correction: multi-scan (*SADABS*; Bruker, 2014)	*k* = −6→6
*T*_min_ = 0.85, *T*_max_ = 0.97	*l* = −21→23
12942 measured reflections	

### Refinement

**Table:**

Refinement on *F*^2^	Primary atom site location: structure-invariant direct methods
Least-squares matrix: full	Secondary atom site location: difference Fourier map
*R*[*F*^2^ > 2σ(*F*^2^)] = 0.060	Hydrogen site location: mixed
*wR*(*F*^2^) = 0.166	H-atom parameters constrained
*S* = 1.05	*w* = 1/[σ^2^(*F*_o_^2^) + (0.0629*P*)^2^ + 1.2077*P*] where *P* = (*F*_o_^2^ + 2*F*_c_^2^)/3
3122 reflections	(Δ/σ)_max_ < 0.001
261 parameters	Δρ_max_ = 0.49 e Å^−^^3^
2 restraints	Δρ_min_ = −0.21 e Å^−^^3^

### Special details

**Table:**

Geometry. All e.s.d.'s (except the e.s.d. in the dihedral angle between two l.s. planes)are estimated using the full covariance matrix. The cell e.s.d.'s are takeninto account individually in the estimation of e.s.d.'s in distances, anglesand torsion angles; correlations between e.s.d.'s in cell parameters are onlyused when they are defined by crystal symmetry. An approximate (isotropic)treatment of cell e.s.d.'s is used for estimating e.s.d.'s involving l.s. planes.
Refinement. Refinement of *F*^2^ against ALL reflections. The weighted *R*-factor *wR* andgoodness of fit *S* are based on *F*^2^, conventional *R*-factors *R* are basedon *F*, with *F* set to zero for negative *F*^2^. The threshold expression of*F*^2^ > σ(*F*^2^) is used only for calculating *R*-factors(gt) *etc*. and isnot relevant to the choice of reflections for refinement. *R*-factors basedon *F*^2^ are statistically about twice as large as those based on *F*, and *R*-factors based on ALL data will be even larger. H-atoms attached to carbonwere placed in calculated positions (C—H = 0.95 - 0.98 Å) while thatattached to oxygen was placed in a location derived from a differencemap and its parameters adjusted to give O—H = 0.84 Å. All wereincluded as riding contributions with isotropic displacementparameters 1.2 - 1.5 times those of the attached atoms. The {N—OH} unitis disordered over two resolved sites in a 3:1 ratio and was refinedsubject to restraints that the geometries of the two components becomparable. The molecule of lattice dioxane located on a center of symmetryappeared to be slightly disordered on the basis of the size and shape ofits displacement ellipsoids but attempts to refine it with a split atommodel were unsuccessful.

### Fractional atomic coordinates and isotropic or equivalent isotropic displacement parameters (Å^2^)

**Table:**

	*x*	*y*	*z*	*U*_iso_*/*U*_eq_	Occ. (<1)
O1	0.31941 (17)	0.8942 (5)	0.31844 (14)	0.0526 (9)	0.761 (4)
H1	0.2653	0.8868	0.3133	0.063*	0.761 (4)
N5	0.3255 (3)	0.7078 (7)	0.27450 (18)	0.0477 (10)	0.761 (4)
O1A	0.2787 (7)	0.6744 (19)	0.2677 (5)	0.0526 (9)	0.239 (4)
H1A	0.2308	0.7294	0.2723	0.063*	0.239 (4)
N5A	0.3523 (7)	0.811 (3)	0.2922 (7)	0.0477 (10)	0.239 (4)
N1	0.50054 (14)	0.0011 (5)	0.10956 (11)	0.0441 (6)	
N2	0.42463 (15)	−0.1296 (5)	0.08969 (12)	0.0483 (7)	
N3	0.54532 (15)	0.3519 (5)	0.17927 (11)	0.0436 (6)	
N4	0.36329 (15)	0.3403 (5)	0.18808 (12)	0.0479 (7)	
C1	0.57561 (18)	−0.0748 (6)	0.08673 (14)	0.0434 (7)	
C2	0.65086 (19)	0.0591 (6)	0.10022 (16)	0.0525 (8)	
H2	0.6541	0.2027	0.1257	0.063*	
C3	0.7218 (2)	−0.0197 (6)	0.07592 (17)	0.0568 (9)	
H3	0.7737	0.0718	0.0849	0.068*	
C4	0.7184 (2)	−0.2271 (6)	0.03920 (15)	0.0529 (9)	
H4	0.7674	−0.2792	0.0229	0.063*	
C5	0.6431 (2)	−0.3581 (6)	0.02643 (16)	0.0558 (9)	
H5	0.6399	−0.5020	0.0011	0.067*	
C6	0.5719 (2)	−0.2820 (6)	0.05014 (15)	0.0526 (8)	
H6	0.5201	−0.3738	0.0410	0.063*	
C7	0.36423 (19)	−0.0231 (6)	0.11490 (14)	0.0478 (8)	
C8	0.27394 (18)	−0.1199 (6)	0.10347 (16)	0.0564 (9)	
H8A	0.2341	−0.0060	0.0763	0.085*	
H8B	0.2573	−0.1429	0.1465	0.085*	
H8C	0.2713	−0.2741	0.0801	0.085*	
C9	0.39917 (18)	0.1795 (6)	0.15255 (14)	0.0457 (8)	
C10	0.48730 (18)	0.1895 (6)	0.14854 (14)	0.0435 (7)	
C11	0.50897 (18)	0.5059 (6)	0.21488 (14)	0.0436 (7)	
C12	0.41977 (18)	0.4993 (6)	0.21947 (14)	0.0467 (8)	
C13	0.40452 (19)	0.6952 (6)	0.26329 (15)	0.0501 (8)	
C14	0.4877 (2)	0.8210 (6)	0.28446 (14)	0.0492 (8)	
C15	0.55051 (18)	0.7031 (6)	0.25596 (14)	0.0455 (8)	
C16	0.6360 (2)	0.7815 (6)	0.26910 (16)	0.0531 (8)	
H16	0.6783	0.7027	0.2500	0.064*	
C17	0.6576 (2)	0.9791 (6)	0.31112 (16)	0.0573 (9)	
H17	0.7157	1.0349	0.3212	0.069*	
C18	0.5955 (2)	1.0949 (6)	0.33833 (16)	0.0595 (9)	
H18	0.6117	1.2305	0.3663	0.071*	
C19	0.5100 (2)	1.0176 (6)	0.32565 (16)	0.0560 (9)	
H19	0.4681	1.0978	0.3448	0.067*	
C20	0.4575 (3)	0.6668 (10)	0.4576 (3)	0.1110 (18)	
H20A	0.4427	0.8360	0.4454	0.133*	
H20B	0.4296	0.5635	0.4198	0.133*	
O6	0.42711 (19)	0.6056 (7)	0.51479 (14)	0.1018 (11)	
C22	0.4493 (3)	0.3649 (12)	0.5297 (3)	0.155 (3)	
H22A	0.4217	0.2617	0.4919	0.186*	
H22B	0.4275	0.3149	0.5696	0.186*	

### Atomic displacement parameters (Å^2^)

**Table:**

	*U*^11^	*U*^22^	*U*^33^	*U*^12^	*U*^13^	*U*^23^
O1	0.0353 (15)	0.068 (2)	0.0564 (18)	0.0043 (13)	0.0138 (13)	−0.0102 (15)
N5	0.042 (3)	0.062 (3)	0.039 (2)	0.007 (2)	0.009 (2)	−0.0014 (18)
O1A	0.0353 (15)	0.068 (2)	0.0564 (18)	0.0043 (13)	0.0138 (13)	−0.0102 (15)
N5A	0.042 (3)	0.062 (3)	0.039 (2)	0.007 (2)	0.009 (2)	−0.0014 (18)
N1	0.0379 (13)	0.0535 (16)	0.0418 (14)	−0.0011 (12)	0.0104 (11)	0.0027 (13)
N2	0.0416 (14)	0.0578 (17)	0.0450 (15)	−0.0042 (13)	0.0081 (11)	0.0065 (13)
N3	0.0394 (13)	0.0542 (17)	0.0378 (13)	0.0035 (12)	0.0095 (11)	0.0052 (12)
N4	0.0387 (14)	0.0641 (18)	0.0424 (14)	0.0025 (13)	0.0122 (11)	0.0115 (13)
C1	0.0427 (17)	0.051 (2)	0.0376 (16)	0.0041 (15)	0.0102 (13)	0.0083 (15)
C2	0.0440 (18)	0.057 (2)	0.058 (2)	−0.0012 (16)	0.0163 (15)	−0.0074 (17)
C3	0.0435 (18)	0.066 (2)	0.062 (2)	−0.0022 (17)	0.0132 (16)	−0.0082 (19)
C4	0.0480 (19)	0.063 (2)	0.0496 (19)	0.0088 (17)	0.0157 (15)	0.0043 (17)
C5	0.060 (2)	0.056 (2)	0.055 (2)	−0.0002 (17)	0.0187 (16)	−0.0035 (17)
C6	0.0488 (19)	0.059 (2)	0.0521 (19)	−0.0069 (16)	0.0157 (15)	−0.0022 (17)
C7	0.0431 (17)	0.063 (2)	0.0383 (17)	−0.0018 (16)	0.0094 (13)	0.0092 (16)
C8	0.0398 (17)	0.073 (2)	0.056 (2)	−0.0058 (16)	0.0100 (15)	0.0081 (18)
C9	0.0394 (16)	0.060 (2)	0.0392 (17)	0.0029 (15)	0.0112 (13)	0.0092 (16)
C10	0.0408 (16)	0.054 (2)	0.0361 (16)	0.0024 (15)	0.0092 (13)	0.0091 (15)
C11	0.0417 (16)	0.054 (2)	0.0365 (16)	0.0077 (15)	0.0113 (13)	0.0096 (15)
C12	0.0387 (17)	0.063 (2)	0.0408 (17)	0.0066 (16)	0.0130 (13)	0.0126 (16)
C13	0.0441 (18)	0.067 (2)	0.0418 (17)	0.0122 (16)	0.0145 (14)	0.0101 (16)
C14	0.0556 (19)	0.058 (2)	0.0350 (16)	0.0144 (17)	0.0121 (14)	0.0073 (16)
C15	0.0418 (17)	0.055 (2)	0.0402 (17)	0.0032 (15)	0.0096 (13)	0.0062 (15)
C16	0.0503 (19)	0.061 (2)	0.0486 (19)	0.0069 (17)	0.0123 (15)	0.0064 (17)
C17	0.054 (2)	0.065 (2)	0.051 (2)	−0.0013 (18)	0.0082 (16)	0.0026 (18)
C18	0.071 (2)	0.061 (2)	0.0463 (19)	0.0031 (19)	0.0110 (17)	−0.0026 (17)
C19	0.061 (2)	0.062 (2)	0.0458 (19)	0.0103 (18)	0.0144 (16)	0.0056 (17)
C20	0.078 (3)	0.147 (5)	0.113 (4)	0.030 (3)	0.032 (3)	0.062 (4)
O6	0.089 (2)	0.151 (3)	0.0728 (18)	0.054 (2)	0.0329 (16)	0.009 (2)
C22	0.082 (4)	0.200 (7)	0.191 (6)	0.047 (4)	0.051 (4)	0.130 (6)

### Geometric parameters (Å, º)

**Table:**

O1—N5	1.390 (4)	C8—H8A	0.9800
O1—H1	0.8400	C8—H8B	0.9800
N5—C13	1.318 (5)	C8—H8C	0.9800
O1A—N5A	1.392 (12)	C9—C10	1.413 (4)
O1A—H1A	0.8402	C11—C12	1.433 (4)
N5A—C13	1.286 (12)	C11—C15	1.453 (4)
N1—C10	1.360 (4)	C12—C13	1.464 (4)
N1—N2	1.389 (3)	C13—C14	1.472 (4)
N1—C1	1.427 (3)	C14—C19	1.382 (4)
N2—C7	1.317 (4)	C14—C15	1.414 (4)
N3—C11	1.331 (4)	C15—C16	1.391 (4)
N3—C10	1.346 (4)	C16—C17	1.394 (5)
N4—C12	1.324 (4)	C16—H16	0.9500
N4—C9	1.351 (4)	C17—C18	1.385 (4)
C1—C6	1.370 (4)	C17—H17	0.9500
C1—C2	1.382 (4)	C18—C19	1.389 (5)
C2—C3	1.389 (4)	C18—H18	0.9500
C2—H2	0.9500	C19—H19	0.9500
C3—C4	1.373 (5)	C20—O6	1.398 (5)
C3—H3	0.9500	C20—C22^i^	1.452 (6)
C4—C5	1.374 (4)	C20—H20A	0.9900
C4—H4	0.9500	C20—H20B	0.9900
C5—C6	1.382 (4)	O6—C22	1.403 (6)
C5—H5	0.9500	C22—C20^i^	1.452 (6)
C6—H6	0.9500	C22—H22A	0.9900
C7—C9	1.412 (4)	C22—H22B	0.9900
C7—C8	1.497 (4)		
			
N5—O1—H1	95.1	N3—C11—C12	124.3 (3)
C13—N5—O1	110.4 (4)	N3—C11—C15	127.5 (3)
N5A—O1A—H1A	117.7	C12—C11—C15	108.2 (3)
C13—N5A—O1A	97.3 (9)	N4—C12—C11	123.9 (3)
C10—N1—N2	110.3 (2)	N4—C12—C13	127.9 (3)
C10—N1—C1	131.4 (3)	C11—C12—C13	108.2 (3)
N2—N1—C1	118.4 (2)	N5A—C13—C12	149.3 (6)
C7—N2—N1	107.5 (3)	N5—C13—C12	115.5 (3)
C11—N3—C10	111.0 (2)	N5A—C13—C14	104.2 (6)
C12—N4—C9	112.8 (2)	N5—C13—C14	138.0 (3)
C6—C1—C2	120.0 (3)	C12—C13—C14	106.5 (2)
C6—C1—N1	119.0 (3)	C19—C14—C15	120.5 (3)
C2—C1—N1	120.9 (3)	C19—C14—C13	130.9 (3)
C1—C2—C3	118.9 (3)	C15—C14—C13	108.6 (3)
C1—C2—H2	120.6	C16—C15—C14	120.8 (3)
C3—C2—H2	120.6	C16—C15—C11	130.6 (3)
C4—C3—C2	121.4 (3)	C14—C15—C11	108.6 (3)
C4—C3—H3	119.3	C15—C16—C17	118.0 (3)
C2—C3—H3	119.3	C15—C16—H16	121.0
C3—C4—C5	118.9 (3)	C17—C16—H16	121.0
C3—C4—H4	120.6	C18—C17—C16	120.8 (3)
C5—C4—H4	120.6	C18—C17—H17	119.6
C4—C5—C6	120.5 (3)	C16—C17—H17	119.6
C4—C5—H5	119.8	C17—C18—C19	121.6 (3)
C6—C5—H5	119.8	C17—C18—H18	119.2
C1—C6—C5	120.4 (3)	C19—C18—H18	119.2
C1—C6—H6	119.8	C14—C19—C18	118.3 (3)
C5—C6—H6	119.8	C14—C19—H19	120.9
N2—C7—C9	109.9 (3)	C18—C19—H19	120.9
N2—C7—C8	121.4 (3)	O6—C20—C22^i^	109.5 (4)
C9—C7—C8	128.6 (3)	O6—C20—H20A	109.8
C7—C8—H8A	109.5	C22^i^—C20—H20A	109.8
C7—C8—H8B	109.5	O6—C20—H20B	109.8
H8A—C8—H8B	109.5	C22^i^—C20—H20B	109.8
C7—C8—H8C	109.5	H20A—C20—H20B	108.2
H8A—C8—H8C	109.5	C20—O6—C22	107.6 (4)
H8B—C8—H8C	109.5	O6—C22—C20^i^	110.7 (5)
N4—C9—C7	131.5 (3)	O6—C22—H22A	109.5
N4—C9—C10	122.4 (3)	C20^i^—C22—H22A	109.5
C7—C9—C10	106.1 (3)	O6—C22—H22B	109.5
N3—C10—N1	128.2 (3)	C20^i^—C22—H22B	109.5
N3—C10—C9	125.6 (3)	H22A—C22—H22B	108.1
N1—C10—C9	106.2 (3)		
			
C10—N1—N2—C7	0.9 (3)	C15—C11—C12—N4	−179.8 (3)
C1—N1—N2—C7	−178.2 (2)	N3—C11—C12—C13	179.6 (3)
C10—N1—C1—C6	176.7 (3)	C15—C11—C12—C13	0.5 (3)
N2—N1—C1—C6	−4.5 (4)	O1A—N5A—C13—C12	0 (2)
C10—N1—C1—C2	−4.3 (5)	O1A—N5A—C13—C14	179.8 (8)
N2—N1—C1—C2	174.6 (3)	O1—N5—C13—C12	177.9 (3)
C6—C1—C2—C3	0.2 (5)	O1—N5—C13—C14	0.6 (6)
N1—C1—C2—C3	−178.8 (3)	N4—C12—C13—N5A	0.6 (15)
C1—C2—C3—C4	−0.2 (5)	C11—C12—C13—N5A	−179.8 (14)
C2—C3—C4—C5	0.1 (5)	N4—C12—C13—N5	2.7 (5)
C3—C4—C5—C6	0.1 (5)	C11—C12—C13—N5	−177.7 (3)
C2—C1—C6—C5	−0.1 (5)	N4—C12—C13—C14	−179.2 (3)
N1—C1—C6—C5	179.0 (3)	C11—C12—C13—C14	0.4 (3)
C4—C5—C6—C1	0.0 (5)	N5A—C13—C14—C19	0.2 (8)
N1—N2—C7—C9	−0.5 (3)	N5—C13—C14—C19	−2.6 (7)
N1—N2—C7—C8	−179.4 (3)	C12—C13—C14—C19	−180.0 (3)
C12—N4—C9—C7	176.7 (3)	N5A—C13—C14—C15	178.9 (7)
C12—N4—C9—C10	−1.9 (4)	N5—C13—C14—C15	176.2 (4)
N2—C7—C9—N4	−178.8 (3)	C12—C13—C14—C15	−1.2 (3)
C8—C7—C9—N4	0.0 (5)	C19—C14—C15—C16	0.4 (4)
N2—C7—C9—C10	−0.1 (3)	C13—C14—C15—C16	−178.5 (3)
C8—C7—C9—C10	178.8 (3)	C19—C14—C15—C11	−179.5 (3)
C11—N3—C10—N1	−178.0 (3)	C13—C14—C15—C11	1.6 (3)
C11—N3—C10—C9	0.1 (4)	N3—C11—C15—C16	−0.3 (5)
N2—N1—C10—N3	177.4 (3)	C12—C11—C15—C16	178.8 (3)
C1—N1—C10—N3	−3.6 (5)	N3—C11—C15—C14	179.6 (3)
N2—N1—C10—C9	−1.0 (3)	C12—C11—C15—C14	−1.3 (3)
C1—N1—C10—C9	178.0 (3)	C14—C15—C16—C17	0.0 (4)
N4—C9—C10—N3	1.1 (5)	C11—C15—C16—C17	179.9 (3)
C7—C9—C10—N3	−177.8 (3)	C15—C16—C17—C18	−0.7 (5)
N4—C9—C10—N1	179.5 (3)	C16—C17—C18—C19	0.8 (5)
C7—C9—C10—N1	0.6 (3)	C15—C14—C19—C18	−0.2 (4)
C10—N3—C11—C12	−0.2 (4)	C13—C14—C19—C18	178.4 (3)
C10—N3—C11—C15	178.7 (3)	C17—C18—C19—C14	−0.4 (5)
C9—N4—C12—C11	1.8 (4)	C22^i^—C20—O6—C22	−59.7 (7)
C9—N4—C12—C13	−178.7 (3)	C20—O6—C22—C20^i^	60.4 (7)
N3—C11—C12—N4	−0.8 (5)		

Symmetry code: (i) −*x*+1, −*y*+1, −*z*+1.

### Hydrogen-bond geometry (Å, º)

**Table:**

*D*—H···*A*	*D*—H	H···*A*	*D*···*A*	*D*—H···*A*
O1—H1···N4^ii^	0.84	2.04	2.878 (3)	172
O1a—H1a···N4^ii^	0.84	1.94	2.749 (5)	162

Symmetry code: (ii) −*x*+1/2, *y*+1/2, −*z*+1/2.
