# Supplementary figures and images for: Crystal structure of N-[(8E)-12-methyl-14-phenyl-10,13,14,16-tetra­aza­tetra­cyclo­[7.7.0.02,7.011,15]hexa­deca-1(16),2,4,6,9,11(15),12-heptaen-8-yl­idene]hydroxyl­amine 1,4-dioxane hemisolvate
Source: Acta Crystallogr E Crystallogr Commun. 2015 Jan 3;71(Pt 2):o75–6. doi: 10.1107/S2056989014027285 (PMC4384549; doi:10.1107/S2056989014027285)

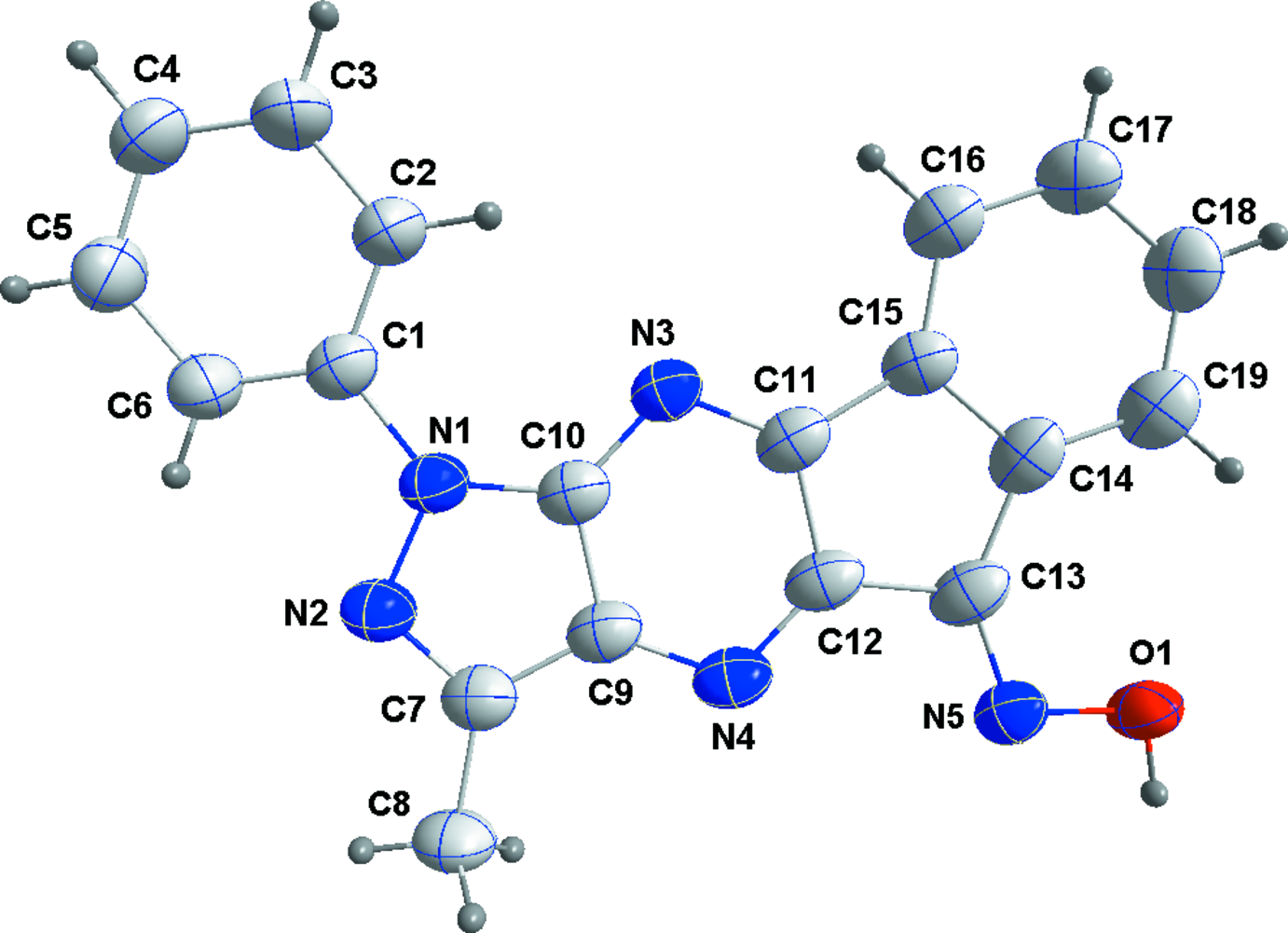

Supplement: Supplementary file 4 [file e-71-00o75-fig1.tif]

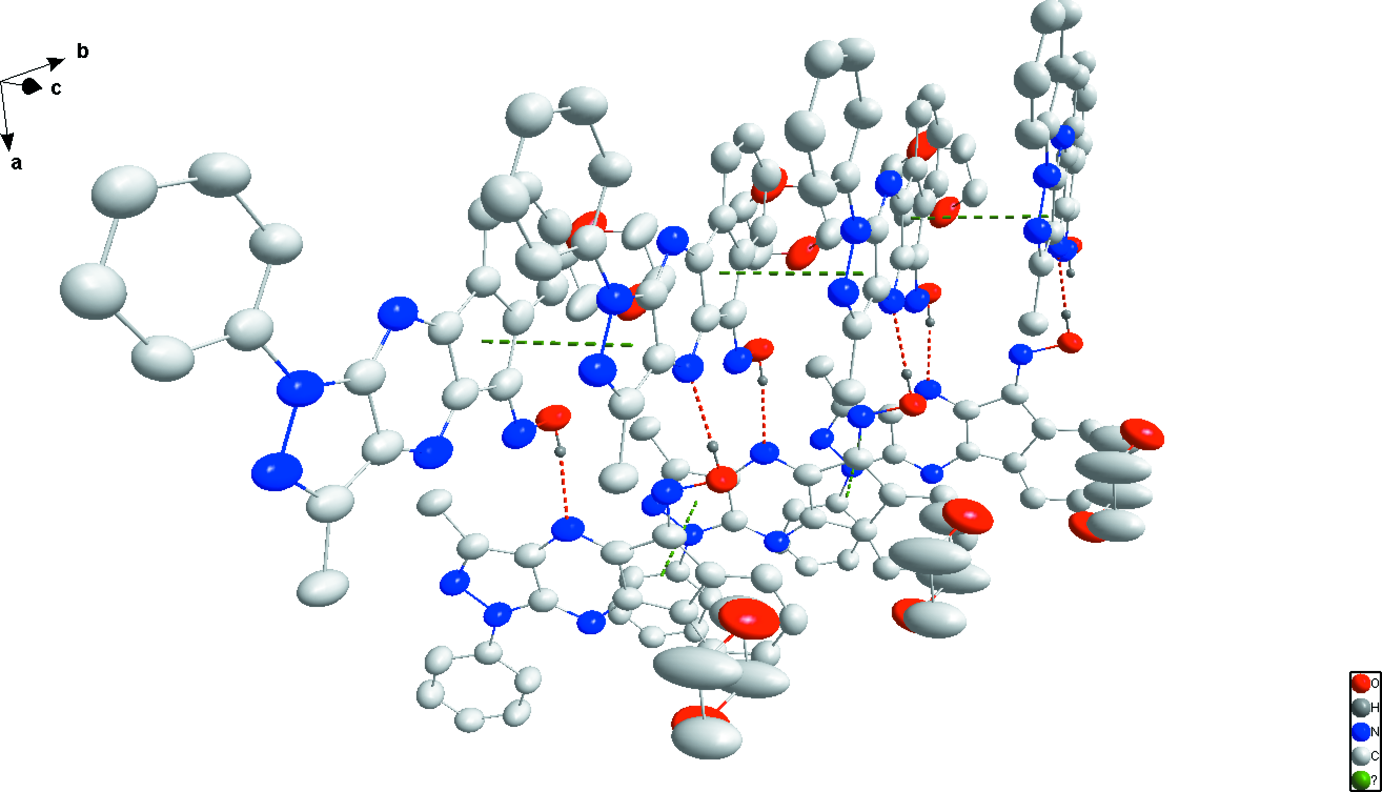

Supplement: Supplementary file 5 [file e-71-00o75-fig2.tif]

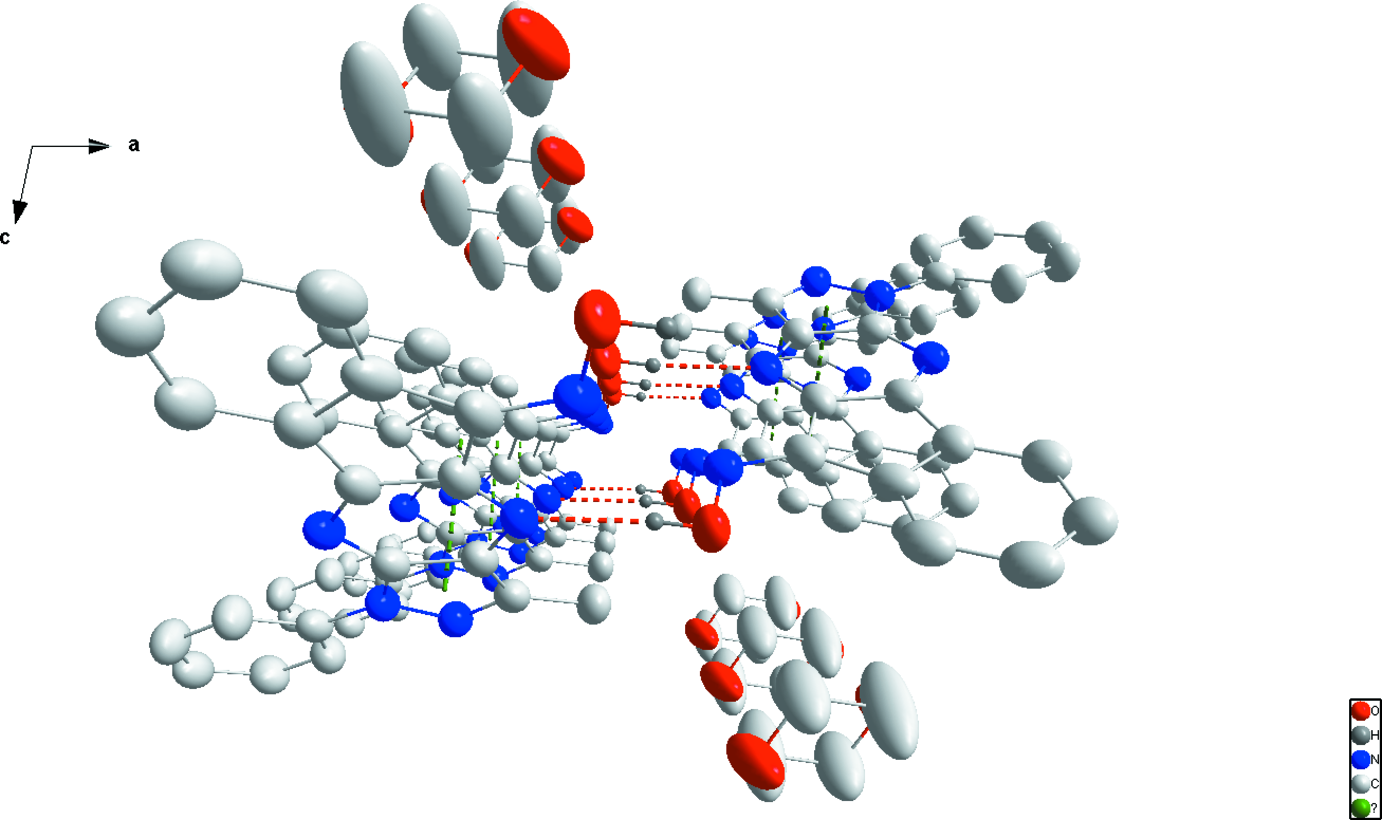

Supplement: Supplementary file 6 [file e-71-00o75-fig3.tif]
